# *In vitro* assays to monitor membrane fusion

**DOI:** 10.3389/fcell.2026.1736159

**Published:** 2026-03-10

**Authors:** Hunter J. Madison, Adam L. Yokom

**Affiliations:** Department of Biochemistry, University of Missouri, Columbia, MO, United States

**Keywords:** *in vitro* reconstitution, membrane fusion, cryo electron tomography, TIRF microscopy, black lipid membranes

## Abstract

Membrane fusion is essential to maintain eukaryotic life. Fusion is tightly regulated and relies on a complex network of protein tethers and lipid interactions. This inherent complexity makes mechanistic investigation of membrane fusion challenging. Taking a reductionist *in vitro* approach has established a fundamental paradigm for most SNARE dependent fusion events. Classically, bulk *in vitro* reconstitution assays leveraging synthetic lipid vesicles can determine the protein assemblies that drive membrane fusion. However, this bulk approach may overlook the heterogeneity of fusion events found within our cells. Recent advancements in single molecule light microscopy and cryo electron tomography enable visualization of individual fusion events at high temporal and spatial resolutions, respectively. In this review we highlight key features of bulk and single fusion assays with a focus on the variables to be considered within each approach. Additionally, we propose potential avenues to expand the *in vitro* toolbox to dissect membrane fusion intermediates.

## Introduction

**FIGURE 1:**
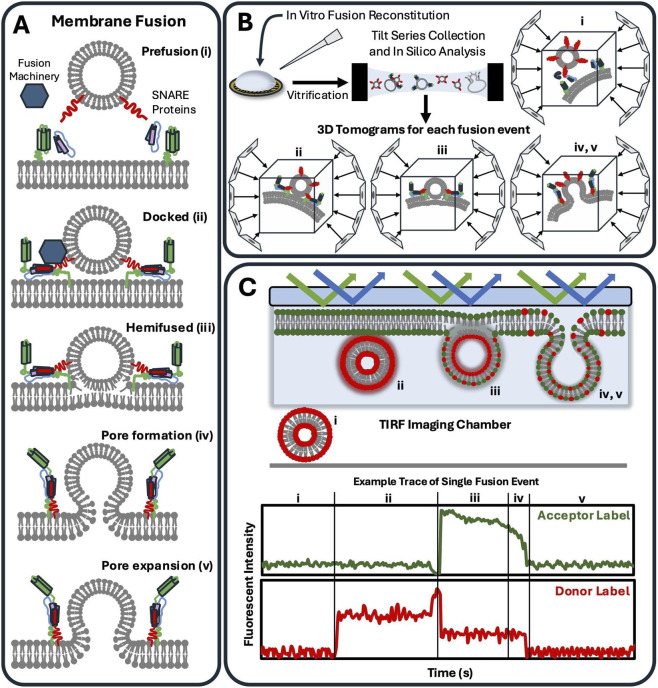
Schematics for membrane fusion, cryo electron tomography and TIRF microscopy **(A)** Distinct states of membrane fusion have been established (Stanton and Hughson, 2023; Wickner, 2010) and shown here as fusion between a vesicle (top) and membrane bilayer (bottom) (i) Initially, fusion machinery is recruited to sites of prefusion on opposing membranes. (ii) After recruitment of effector proteins, membrane bilayers are pulled together in a docked conformation. (iii) Hemifused intermediates are proposed to occur once the energetic barrier of fusion is overcome and the outer leaflets merge. (iv) Finally, inner bilayer leaflets meld together, and a fusion pore is formed which is quickly followed by (v) pore expansion. **(B)** Established *in vitro* membrane fusion reconstitution directly used as starting points for Cryo electron tomography (cryo-ET) experiments. Upon vitrification, high resolution cryo-ET imaging can lead to 3-dimensional data of single fusion events. Leveraging *in silico* analysis, cryo-ET can isolate structural data for fusion intermediates including prefusion (i), docking (ii), hemifusion (iii), and post fusion (iv,v). **(C)** Total internal reflection fluorescence (TIRF) microscopy can be paired with supported lipid bilayers or black lipid membranes to track single and multiple fusion events at high temporal resolution. Imaging conditions can be adapted to separate fusion state intermediates over times ranging from milliseconds to minutes. In the representative trace shown, a FRET pair is incorporated such that the donor fluorophore labels vesicles and acceptor fluorophores label a SLB. In the prefusion state (i), both channels exhibit basal fluorescence, and most vesicles will be out of the imaging plane. Upon vesicle docking (ii) in the evanescent field, donor fluorescence spikes. Hemifusion (iii) enables outer leaflet lipid mixing, resulting in increased FRET efficiency, characterized by elevated acceptor fluorescence and decreased donor fluorescence. Fusion pore formation (iv) and expansion (v) can be detected as a decrease in FRET transfer efficiency as lipids laterally diffuse.

Membranes play an essential role in numerous processes throughout our cells, including organelle compartmentalization, molecular entry regulation, homeostasis and others (Watson, 2015). The breadth of biological functions correspond with sophisticated membrane specialization (Harayama and Riezman, 2018). Lipid composition alone governs fluidity, curvature, peripheral protein binding, and charge distribution (McMahon and Boucrot, 2015; Baccouch et al., 2023). Our membranes and their associated protein complexes interact in a variety of ways, including transient tethering, as seen in organelle contact sites (Pan et al., 2000; Voeltz et al., 2024), regulated lipid exchange (Kovács et al., 2023), and membrane fusion. Membrane fusion is perhaps the most enigmatic of these processes, as melding lipid bilayers together entails overcoming a high energetic barrier and often necessitates many auxiliary factors (Wickner, 2010; Stanton and Hughson, 2023). Each fusion event between bilayers requires dedicated protein assemblies that facilitate a stepwise progression through membrane recognition and docking, stalk formation and hemifusion, and the final expansion and formation of a fusion pore (Chernomordik and Kozlov, 2008; Wickner and Schekman, 2008; Jahn et al., 2024) (Figure 1A). The rate of fusion itself is dependent on the cellular context and is membrane specific (Jahn and Scheller, 2006; Stenmark, 2009; Akimov et al., 2020). Notably, membrane fusion events themselves are rarely symmetric, often incredibly complex, and are essential in a multitude of essential cellular pathways including autophagy, synaptic transmission, viral entry, and the trans-Golgi network (Söllner and Rothman, 1994; Nichols and Pelham, 1998; Harrison, 2008; Upton et al., 2024), to name a few.

Although fusion machineries comprise diverse macromolecular components, the prevailing paradigm has largely been established through mechanistic studies of the factors required to promote membrane fusion (Wickner, 2010) (Figure 1A). Soluble N-ethylmaleimide-sensitive-factor attachment protein receptor (SNARE) proteins are a family of eukaryotic proteins that have become ubiquitously associated with membrane fusion (Jahn et al., 2024; Jahn and Scheller, 2006). Specialized SNARE complexes have been identified for a number of distinct fusion events such as vesicles/organelles, exocytosis, and endocytosis, neurotransmitter vesicle fusion during synaptic release in neurons (Rizo and Rosenmund, 2008; Dash et al., 2025), and autolysosomal fusion between autophagosomes and lysosomes (Ke, 2024). Trans-SNARE complexes are preloaded onto opposing membranes at the site of fusion (Jahn et al., 2024). Individual SNARE proteins are anchored to membrane bilayers and spontaneously form a *trans*-assembly helical bundle that “zippers” the two membranes together, with the help of regulator proteins (Figure 1A). This interaction overcomes the energetic barriers associated with membrane dehydration, phospholipid headgroup repulsion, and transient exposure of lipid hydrophobic tails. Well-known regulators for SNARE-mediated fusion are Sec1/Munc-18 protein family (Südhof and Rothman, 2009; Rizo, 2022), the CATCHR family of tethering complexes (Szentgyörgyi and Spang, 2023), and the Exocyst complex (Maib and Murray, 2022). Though the functionality of SNAREs is conserved across many fusion events, it is certainly not the only fusion-driving assembly.

In contrast to SNAREs, viral fusion is regulated by a single fusogen protein that undergoes large conformational changes in response to stimuli. These fusogen proteins, which notably include proteins such as hemagglutinin from Influenza and the spike protein from SARS-CoV-2, share a “trimer-of-hairpins” that facilitate fusion. Fusogen proteins are essential for viral entry and fitness and highlight the critical importance of membrane fusion in viral infections. In the context of nonviral membrane fusion, misregulation or dysfunction of fusion machinery can lead to significant cellular impairment. Various human diseases and disorders have been attributed to membrane fusion failure, including: neurodegenerative diseases (Hou et al., 2017), cancers (Liu et al., 2024), muscular dystrophies (Dias and Nylandsted, 2021), and lysosomal storage disorders (Fraldi et al., 2010), among others (Zhu et al., 2003; Ranieri et al., 2013).

Due to its widespread biological relevance, the study of membrane fusion has been a focus of many research labs. Decades of research has formed the basis of the membrane fusion paradigms described above (Jahn et al., 2024). However, due to the transient nature and crowded cellular environment, dissecting discrete mechanistic information has proven difficult. To this end, considerable effort has been directed toward establishing *in vitro* assemblies that reconstitute membrane fusion events. These assays aim to reduce the complexity inherent to cellular contexts to gain a mechanistic view of fusion. By stripping down the complexity of fusion, *in vitro* assemblies enable control over lipid composition, membrane geometry, and protein composition. These *in vitro* reconstitutions create a powerful, yet controllable toolbox for dissecting membrane interactions and fusion.

Given that asymmetry inherently influences fusion energy barriers (Shendrik et al., 2025) and the dynamic interactions of proteins and other macromolecules that drive fusion, it has become increasingly clear that fusion events are very heterogeneous. This has prompted methods suitable to resolve single fusion events. In this review we aim to highlight reconstitution approaches in two categories: bulk assays and single fusion events. We focus on detailing advantages and limitations of these approaches which are highlighted in Table 1. Finally, we will discuss future potential for exploring asymmetric fusion events and investigation of prefusion intermediates.

**TABLE 1:** Summary of *in vitro* fusion assays including type of fusion measurement, advantages, and limitations.

Method	Fusion measurement	Advantages	Limitations
Lipid mixing	Bulk	Established, low cost	Dependence on lipid diffusion
Content mixing	Bulk	Established, low cost	Vesicle leaking
Black lipid	Bulk and single fusion events	Solvent accessible, capable of electrophysiology studies and tunable	Fragile, technically challenging and low reconstitution efficiency
Nanodiscs	Single fusion event	Stability, established for structure determination	Purification cost, small membrane surface
SLBs	Bulk and single fusion events	Stability, detect distinct fusion states	More rigid, limited dynamics
Cryo electron tomography	Single fusion event	Spatial resolution, visualize all fusion intermediates	Access, cost, data processing intensive
TIRF	Single and multiple fusion events	Temporal resolution, fusion kinetics	Restricted field of view, technically challenging

## *In Vitro* reconstitutions

### Bulk fusion assays

Bulk fusion assays are the predominate method for studying fusion *in vitro*. Bulk experiments have proven crucial for determining fusion kinetics, identifying which protein complexes drive fusion and isolating how lipid head groups influence fusion. Overall bulk assays balance simplicity with complexity. Bulk assays are relatively high throughput and analysis is straightforward. Bulk workflows begin with formation of membrane vesicles. These synthetic vesicles can range from giant unilamellar vesicles (1–10 μm) to small unilamellar vesicles (<100 nm). Formation can be accomplished with crude extracts of lyophilized lipids or from chemically synthetized lipids.

Bulk fusion assays have many tunable parameters to tailor to each biological question. Vesicle size is one critical parameter that directly influences membrane curvature, which in turn affects lipid packing density and local energetic barriers to fusion. Notably, cellular pathways, such as cell migration (Sitarska et al., 2023) and organelle-membrane contact sites (Yang et al., 2024), depend on sensing membrane curvature and curvature changes influence downstream events. Additionally, bulk assay reconstitutions are often prepared with asymmetric protein complexes prior to vesicle fusion experiments. This practice is well-established in reconstitution studies and relies on reconstituting vesicles with trans-SNAREs or other membrane-associated fusogenic proteins distributed across separate vesicle samples. Additional parameters to consider include the use of synthetic chemical handles (such as protein affinity tags (Elliott and Prestwich, 2000; Schmid et al., 2015; Zhang et al., 2024; Lee et al., 2025), replicating post-translational lipidation pathways *in vitro* (Litschel et al., 2021), and osmotic which influences lipid bilayer stability. Ultimately, there are many parameters which can be explored to enhance bulk fusion assays to target specific biological questions.

Lipid mixing assays rely on tracking fluorescent signals as a proxy for measuring fusion rates of synthetic lipid vesicles. Often incorporation of fluorescently labeled lipid headgroups such as NBD (N-7-nitro-2,1,3-benzoxadiazole-4-yl) or Rho (N-lissamine rhodamine B sulfonyl) into unilamellar vesicles is performed during lipid vesicle formation. Experimentally, NBD and Rho lipids are integrated at low concentrations (0.8%–1.5% of total lipid) which has minimal off target effects (François-Martin and Pincet, 2017). A caveat to this approach is that lipid mixing assays require lipid diffusion to distribute labelled headgroups. Therefore, hemifused intermediates (Figure 1A) could allow lipid diffusion. This nuance may lead to inconclusive data about which protein assemblies drive fusion. Content mixing assays were developed to address this pitfall. Fluorescent labels, such as calcein and sulforhodamine B, are conjugated to dextran inside the lumen of fusion vesicles (Bowen et al., 2004). Only upon pore formation will the concentration of the quenching dye decrease and allow fluorescence. Common quenching concentrations range from 50–200 mM calcein (Bowen et al., 2004) and 20–50 mM sulforhodamine B (Yang J. et al., 2016). Content mixing is limited by the bulk fusion measurement and the possibility that vesicle leaking will dequench encapsulated fluorophores, resulting in false positives for fusion.

An alternative to fluorescent based bulk assays is the use of black lipid membranes (BLMs) also termed as planar or bimolecular lipid membranes. BLMs are ultra-thin, free-standing lipid bilayer mimetics that span across an aperture. This arrangement allows solution access on both sides and electrical conductivity to be monitored. The accessibility of both sides of the membrane bilayer enables BLMs to support diverse and innovative experimental configurations, which have been leveraged to monitor bulk fusion kinetics and, in some instances, single fusion events (the latter is described under TIRF microscopy) (Schwenen et al., 2015; Tsemperouli et al., 2019). Notably, BLMs are fragile and membrane tension can vary across the suspended lipid bilayer. Further details regarding the advantages and limitations of BLMs have recently been described by Coronado et al. (Coronado et al., 2024). Taken together, bulk fusion assays have formed the foundation of *in vitro* quantification of membrane fusion. These approaches lead to many discoveries and played a key role in uncovering the biophysical properties of viral fusogen peptides (Parente et al., 1988). However, bulk assays are not suited to answer longstanding questions about homogeneity of membrane fusion events, including the diversity of protein complexes that are required to promote fusion *in vivo*. Instead, methods to monitor single fusion events are required to explore individual fusion events in greater detail. We will highlight recent developments that enabled the visualization of individual fusion events at higher spatial and temporal resolutions.

## Monitoring single fusion events

Monitoring single fusion events is not a novel concept, and initial attempts employed lipid nanodiscs to characterize fusion assemblies, particularly in the context of SNARE-mediated fusion (Shin et al., 2014; Wu et al., 2016). Nanodiscs are lipid bilayer bundles that are held together by membrane scaffold proteins or amphipathic polymers (Grant and Schmidt-Krey, 2025). Nanodiscs provide a tunable bilayer, like the synthetic vesicles used in bulk fusion assays. However, nanodiscs are limited by their relatively small lipid surface area (10–50 nm in diameter) and contain exclusively discoidal membrane surfaces (Elzoghby et al., 2023). These parameters are mismatched with large, curved membranes found in many biological fusion events. The following section will focus on two novel applications to capture single fusion events: cryo-electron tomography (cryo-ET) and total internal reflection fluorescence (TIRF) microscopy. These approaches have initial drawbacks including access to high end microscopy equipment and technically challenging sample preparation and data analysis. However, both approaches show unprecedented spatial or temporal resolution, respectively.

### Cryo electron tomography

Advancements in cryo-electron tomography (cryo-ET) have shown an extraordinary ability to visualize superstructure assemblies across many fields of biology. The developments in data acquisition, subtomogram averaging and throughput allow dissecting of increasingly complex samples (Nogales and Mahamid, 2024). Typically, cryo-ET is thought of being coupled to focused ion beam milling for *in situ* structure determination. Cryo-ET is equally well suited to visualize *in vitro* membrane fusion events at high resolution (<5Å). Cryo-ET workflows have been described for *in situ* structural biology and can be directly applied to characterization of *in vitro* membrane reconstitutions (Chen et al., 2019). Briefly, reconstituted samples can be vitrified and amorphous ice is formed. These ‘static’ samples are imaged using a high-powered electron microscope and each individual event can be imaged across multiple angles ranging from 0° to ±45° creating a ‘tilt-series’ (Figure 1B) (Wan et al., 2016). Tilt series are aligned and generate 3-dimensional tomograms with subnanometer resolutions (de la Mora et al., 2021). Cryo-ET has the potential to structurally characterize individual fusion intermediates without the need of fluorescent labels. A limitation to this approach is the access and cost of instrumentation and the ability to analyze tomographic data.

High resolution (<10Å) tomography reconstructions have mainly come from supramolecular assemblies that form repeating arrays. This includes protein coats comprised of AP-1:Arf1, COPII, and effector proteins bound to microtubules (Ferro et al., 2022; Hooy et al., 2022; Pang et al., 2025). Most fusion events are not expected to form repeating elements (Maib and Murray, 2022). However, visualizing individual fusion events can help unravel this complexity and identify the superstructure assembly and illuminate how regulator complexes (Sec1/Munc-18 and CATCHR families) are organized during prefusion (i), docking (ii) and fusion (Figure 1A). Finally, cryo-ET can leverage the customization of *in vitro* reconstitutions to delineate prefusion assemblies. Dedicated methods have been developed to identify fusion states on cryo electron microscopy grids (Metskas and Briggs, 2019). Thoughtful design of lipid composition, molecular components and goals of each experiment should be considered before investments are made to acquire cryo-ET data.

### Total internal reflection microscopy

The strength of cryo-ET lies in its exceptional spatial resolution, but it remains dependent on static snapshots of fusion events. Total internal reflection fluorescence (TIRF) microscopy is an alternative method to visualize single fusion events. This allows real-time, dynamic quantification of fusion events (Domanska et al., 2009). By utilizing the properties of standing evanescent waves during total internal reflection, laser excitation is restricted to a very thin region (<200 nm), which drastically reduces background fluorescence and allows quantification of local fluorescent signals (Figure 1C). Förster Resonance Energy Transfer (FRET) fluorophore pairs, when coupled with TIRF microscopy, can be used to discern vesicle and lipid bilayers during prefusion, docking, fusion and pore expansion with real time perceptible fluorescence changes. From individual tracks, fusion events can be segmented into fusion intermediates such as docked (ii), hemifused (iii) or post fusion (iv, v) (Figure 1C). TIRF microscopy can be paired with planar membranes methods such as supported lipid bilayer (SLBs) and BLMs. SLBs are deposited on coverslips and allow tracking of individual fusion events across a large lipid bilayer surface (Nikolaus et al., 2021). Only the labeled vesicles which dock to the planar membrane are illuminated, which allows sensitive and temporal resolution of discrete binding and fusion events for determination of fusion kinetics. Unique to TIRF microscopy is its ability to detect sequential events over a given time course. BLMs can be generated on horizontal platforms which maintain optical imaging capabilities (Schwenen et al., 2015; Tsemperouli et al., 2019). Furthermore, planar membrane assemblies can be functionalized with established lipid modification methods (Dam et al., 2022). Lastly, it is worth noting that microfluidics has also been coupled to *in vitro* reconstitution design to allow for increased control and precision in reagent delivery (Kim et al., 2024).

The field of viral fusion was initially established using bulk fusion assays and is now an early adopter of both cryo-ET and TIRF microscopy approaches. Recent work has captured heterogeneous assemblies of viral fusion intermediates (Kephart et al., 2024; Akıl et al., 2025) albeit at moderate resolutions. These studies utilized a combination of *in vitro* reconstitution and tomography data to understand the general principles of viral fusion. These studies showed prefusion, closely docked and fused states. These cryo-ET approaches used for viral fusion are directly transferrable to investigation of membrane fusion in other systems. The combination of TIRF and giant plasma membrane vesicles was leveraged to investigate how SERINC homologs, which incorporate serine into membrane lipids, promote fusion (Ward et al., 2020). This approach was critical in understanding the defects in content-release of the viral particle inhibitor, amphotericin B, which did not disrupt binding, but significantly slowed pore formation (Ward et al., 2020). Similarly, TIRF imaging with specialized SLBs showed how HIV fusogens preferentially drive fusion at interfaces between ordered and disordered lipid regions (Yang ST. et al., 2016). This highlighted the role of line tension and the organization of lipid rafts in HIV peptide-mediated fusion (Yang ST. et al., 2016). SNARE dependent fusion events can be explored using analogous methods to reveal the temporal parameters between docking and fusion under a variety of conditions. Overall, the use of cryo-ET and TIRF to investigate single fusion events will deepen our understanding of questions which are inaccessible using bulk assays.

## Future perspectives

*In vitro* membrane assemblies continue to be a valuable tool at researchers’ disposal to create tunable experiments for addressing discrete, mechanistic questions of biological importance. Advancements have enabled dissection of single fusion events at high spatial and temporal resolutions. Experimental designs have similarly evolved to provide enhanced suitability for various biological questions. As the field of membrane fusion continues to develop, it is important to explore novel methods to advance our understanding of this complex and essential cellular process.

Recent work has begun to adapt bulk fusion assays to monitor upstream docking and tethering to isolate prefusion intermediates. From fluorescent labels to geometric and molecular asymmetric design, these *in vitro* systems can dissect biophysical principles of membrane fusion. Notably prefusion states may be present in cryo-ET datasets where hundreds to thousands of individual fusion events are frozen. A unique alternative to capturing the spontaneous docking and tethering events within a bulk sample is utilizing DNA-lipid tethers (Marie et al., 2023). DNA-lipid tethers have recently been shown capable of generating a functionalized membrane surface which can be tethered through direct DNA-DNA interactions. DNA-lipid tethers utilize base complementarity to bypass the need for protein functionalization while still allowing experimental control in driving fusion. This synthetic strategy is proposed as a potential mode of liposome-encased drug delivery (Armistead et al., 2023). Furthermore, DNA-lipid tethers experiments have shown that classical cell-cell adhesion follows fundamentally different physical principles as compared to organelle contact formation mediated by cytosolic tethers (Kamal et al., 2022).

Another avenue to be explored and coupled to *in vitro* reconstitutions is the development of asymmetric systems that are closer to physiological fusion events. This can be done through a few unique approaches. Firstly, vesicles can be built with lipids which favor unique morphologies beyond the typically sphere shape. A recent study by de la Mora et al. leveraged galactocerebroside lipids to form tubular vesicles as a functional replacement for a spherical vesicle formation (Di Cicco et al., 2025). Galactocerebrosides are a main component of neuronal myelin sheath and spontaneously form tubular structures. This feature was paired with cryo-ET to resolve how VAP-A, an ER transmembrane protein, facilitates membrane contact sites between apposed organelle membranes (de la Mora et al., 2021). Given that a bottle neck in cryo-ET data analysis is initial particle identification, the asymmetric vesicle shape aided in determining the directionality of the protein-lipid interface. While it has not be shown that galactocerebrosides can undergo complete fusion, it is intriguing to explore experiments that exploit the tubular structure. Overall, it is an exciting time where the combination of established bulk assays and developing methods to capture individual fusion events is likely to bring novel insights into membrane fusion mechanisms.
